# Seizure-Related Head Injuries: A Narrative Review

**DOI:** 10.3390/brainsci14050473

**Published:** 2024-05-08

**Authors:** Sebastian Piwowarczyk, Paweł Obłój, Łukasz Janicki, Kornelia Kowalik, Adam Łukaszuk, Mariusz Siemiński

**Affiliations:** 1 Department of Emergency Medicine, Medical University of Gdansk, Mariana Smoluchowskiego 17, 80-952 Gdansk, Poland; s.piwowarczyk@gumed.edu.pl (S.P.); paobloj@gmail.com (P.O.); lukasz.janicki@gumed.edu.pl (Ł.J.); kornelia.kowalik@gumed.edu.pl (K.K.); 2 Edinburgh Medical School, College of Medicine And Veterinary Medicine, The University of Edinburgh, Edinburgh EH8 9YL, UK; a.z.lukaszuk@sms.ed.ac.uk

**Keywords:** epilepsy, seizures, head injury, traumatic brain injury

## Abstract

Epilepsy is one of the most common neurological diseases. Epileptic seizures very often result in head injuries that may lead to many adverse consequences, both acute and chronic. They contribute to the need for hospitalization, modification of treatment, and a general decline in social productivity. The objective of our review is to characterize and assess management aspects of seizure-related head injuries (SRHIs) as an important and frequent clinical problem present in emergency department settings. PubMed and other relevant databases and websites were systematically searched for articles on traumatic brain injuries connected with the occurrence of seizures published from inception to 9 April 2024; then, we reviewed the available literature. Our review showed that SRHIs can lead to various acute complications, in some cases requiring hospitalization and neurosurgical intervention. Long-term complications and cognitive decline after injury might be present, eventually implying a negative impact on a patient’s quality of life. Despite being frequent and clinically important, there are still no widely accepted, uniform recommendations for the management of patients with SRHIs. As such, a concise and standardized protocol for the management of seizure-related head injuries in emergency departments is worth consideration.

## 1. Introduction

Seizure-related head injuries (SRHIs) are typically defined as any dysfunction, impaired consciousness, or pain localized in the head region resulting from accidental occurrence during seizures [1]. Postictal activity in the form of delirium or psychosis may occur after seizures have ended and may have a detrimental effect on the course of treatment in patients with epilepsy (PWE). Both seizure-related head injuries (SRHIs) and postictal activity contribute to direct and indirect medical costs. They contribute to the need for hospitalization, modification of treatment, and a general decline in social productivity [2].

Epilepsy is one of the most common neurological diseases. Epileptic seizures very often result in physical injuries—including head injuries but not only [3]. Seizure-related head injuries lead to many adverse consequences, both acute and chronic. Acute consequences include wounds, concussions, fractures, epidural and subdural hematomas, and intracranial hemorrhages [4]. There is also a complication called post-traumatic syndrome. This covers a wide spectrum of symptoms occurring after a traumatic brain injury (TBI) [5]. Epilepsy patients can be divided into groups, taking into account additional risk factors for head injuries.

Seizure-related head injury is a prevalent medical condition in people with epilepsy that may eventually lead to several different short- and long-term health conditions. Despite being common and potentially difficult to manage, recommendations on the topic are scarce and often ambiguous, especially in the case of mild traumatic brain injuries associated with seizures. The objective of our review was to characterize and assess the management aspect of seizure-related head injuries as an important and frequent clinical problem present in emergency department settings.

## 2. Materials and Methods

PubMed and other relevant databases and related websites in English were systematically searched for articles on “epilepsy”, “head injury”, “seizures”, and “traumatic brain injury” published from inception to 9 April 2024, finding 392,725 entries. Then, the results were filtered using criteria of the date of publication, including studies published in the years 2014–2024, and relevance to the topic of our review, using the presence of combinations of our search queries in study abstracts to search for literature on traumatic brain injuries connected with the occurrence of seizures. Abstracts of the filtered publications were read and, after meeting the relevance and quality criteria, reviewed thoroughly after reading the whole publications; 29 studies were included. Considering the limited number of studies on certain aspects of our review, 11 studies of adequate quality published outside our targeted timeframe were also included. Altogether, 40 articles were included in our review.

## 3. Epidemiology

Seizure-related head injuries (SRHIs) are typically defined as any dysfunction, impaired consciousness, or pain localized in the head region resulting from an accidental occurrence during seizures [1]. There are a few injury severity classifications of seizure-related head injuries proposed throughout the literature. One of the most prevalent criteria defines mild SRHI as consciousness impairment lasting less than 30 min with no associated skull fracture, moderate as at least 30 min up to 24 h of impaired consciousness or skull fracture, and severe injury is classified as amnesia or consciousness impairment lasting more than 24 h, a present brain contusion, or intracranial hematoma [1,6]. Other authors use more simplistic approaches, such as utilizing the Glasgow Coma Scale—proposed specifically to assess head trauma. They classify head injury severity based on the GCS score alone: mild injury is defined as a GCS score ranging from 15 to 13, moderate as GCS 12–9, and severe as a GCS score below 9 [7,8].

Epilepsy is one of the most common neurological disorders. The exact prevalence of a condition is difficult to estimate as data differs significantly among countries, ethnicities, and social backgrounds. Consequently, the demographic structure of the study population impacts the results substantially. In a systematic review and meta-analysis of a total of 222 studies on epilepsy incidence, the lifetime prevalence of epilepsy was estimated to be 7.60 per 1000 people [9]. Considering the additional risk factors of head injuries in adults, two separate groups of elevated hazards emerge. The first group—young adults with increased head trauma risk from motor vehicle accidents—is an issue of concern for people with epilepsy [10]. The second group involves people with numerous comorbidities in addition to known fall-risk-increasing drugs [11] and standard epilepsy-related balance and mobility alteration [12], resulting in falls, eventually putting them at risk of head injuries. Epidemiological studies show that epilepsy incidence rates are higher in the youngest and the oldest age groups—86 per 100,000 in the first year of age with a subsequent decrease, stabilizing around the age of ten, up until the age of 54 when an increase occurs. As such, the young adult cohort has the lowest incidence of seizures in all the age groups—23–31 per 100,000 in the 30–59 age group, while incidence rates tend to increase after the 54th year of life, reaching 180 per 100,000 in the 85+ cohort [13].

Epileptic seizures frequently result in distinct physical injuries, fractures, traumatic brain injuries, and minor trauma. Data on the frequency and extent of seizure-related injuries are inconclusive and, unfortunately, there are no adequate studies on large cohorts [3]. A review of the literature shows large discrepancies in the statistics regarding typical injuries and their severity. These differences result, of course, from the groups of patients on which individual studies focused, but also from the method of collecting and analyzing the data.

A study conducted in 2022 in Saudi Arabia showed that the most common injuries reported were soft tissue injuries (36.5%) and head trauma (32%). Other types of injury reported in the study included: dental injuries (8.5%), burns (7%), and dislocations (7%). Furthermore, 6.5% of the patients had fractures, and 2% of the patients experienced submersion [14]. Worth noting here is the self-reporting nature of the study: aside from reviewing patients’ medical records, the data were acquired by interviewing participants utilizing a structured questionnaire. The patients and accompanying relatives were surveyed by researchers using a prepared questionnaire.

In comparison, a 2021 study conducted on a group of 94 patients showed that seizure-related injuries could be partitioned into fractures (48.9%), craniocerebral trauma (26.6%), and soft tissue injuries (24.5%). Of the soft tissue injuries in the studied population, the most prevalent were lacerations, representing 11.7% of all injuries reported regardless of type, and then as follows: luxations (6.4%), sprains (4.3%), and cuts and compartment syndromes (each 1.1%). Regarding craniocerebral trauma, injuries reported were concussion—representing 68.0% of the aforementioned category of injury, followed by cerebral contusion (20.0%), and brain compression (8.0%). Only one case of subdural hemorrhage (4.0% of reported head traumas) was diagnosed as a seizure-related injury, and it was associated with a motor vehicle accident [3]. In this study, a retrospective, systematic query of a hospital information system was performed. Completely different from the publication cited above, which directly translates into injury statistics, it is important to note that this retrospective analysis was conducted in a tertiary care hospital and a national trauma center with an associated national epilepsy center, as well as in a large combined rural and metropolitan area that admits more than 320,000 patients annually. This undoubtedly affects the profile of the patients admitted in this unit, and thus also the study group presented in this publication.

Children are a special group of patients who should be analyzed separately for seizure-related injuries. A retrospective study from 2019 showed completely different statistics regarding the complications of traumatic seizures. It showed that of all injuries, 30% were lacerations that required suturing, 19% were fractures, 14% were dental injuries, 10% were concussions, 5% were burns, and 25% were labeled as “other”. The “other” category consisted of one fatal drowning, two near-drownings, three shoulder dislocations, and one severe eye trauma [15]. In this study, the authors included a population consisting of residents of the Province of Nova Scotia, meeting the inclusion criteria of being between 1 month and 16 years of age at the time of epilepsy onset and who developed epilepsy between 1977 and 1985. The authors defined epilepsy as at least two unprovoked episodes of seizures. Not all epileptic patients meeting the aforementioned criteria were recruited in the study; childhood absence epilepsy patients were excluded by the authors, as they studied that specific population in different research. Progressive brain disorders, for example, malignant brain tumors, neurodegenerative diseases, and metabolic diseases were also used as exclusion criteria during recruitment of the studied population.

A review of the available literature does not provide a clear answer to questions regarding the statistics of head injuries related to seizures. The available data cover various types of complications of epileptic seizures—including traumatic complications. It is necessary to conduct further research and statistical analyses on head injuries, divided into specific types of injuries. This could include isolated data on head injuries secondary to a seizure, with an additional division into soft tissue injuries, skull injuries, and primarily a CNS complication, defined as the presence of fresh blood in the CNS or a midline shift. The results of these studies could have a direct impact on the possibility of use in clinical practice.

According to the Centers for Disease Control and Prevention (CDC) definition, a TBI is caused by a bump, blow, or jolt to the head, or a penetrating head injury that disrupts the normal function of the brain. Traumatic impact injuries can be defined as closed (nonpenetrating) or open (penetrating) [16]. In analyzing cases of TBIs, most of them are mild (GCS between 13 and 15), with the frequency estimated at around 80%. Approximately 10% of TBIs are of moderate severity, with GCS ranging from 9 to 12, and about 10% are classified as severe TBIs, with GCS from 3 up to 8. Approximately 1.1 million of the TBIs are mild. The incidence of non-mild TBIs is estimated to be 15 cases per 100,000 people in the case of moderate TBIs, and 14 cases per 100,000 people for severe ones. Unsurprisingly, mortality deviates towards TBIs of higher severity. Generally speaking, the out-of-hospital TBI mortality rate is 17 per 100,000 and decreases to 6 per 100,000 in the case of in-hospital ones [8]. A moderate or severe TBI is caused by a bump, blow, or jolt to the head or by a penetrating injury (for example: from a gunshot) to the head. In the United States, thousands of deaths each year are associated with severe TBIs [17]. From a clinical point of view, from the perspective of an ED doctor, the most important are mild traumatic brain injuries (mTBIs). Although patients with moderate or severe TBIs constitute a group with the highest potential threat to life, patients presenting symptoms of an mTBI, due to their number and sometimes discreet symptoms, constitute a very difficult group to diagnose. Caring for this group of patients requires extremely high attention and vigilance from the clinician.

**Figure 1:**
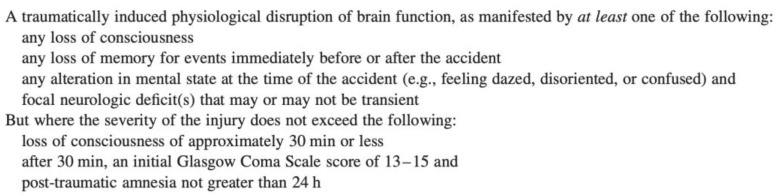
Diagnostic criteria for mild TBI by the American Congress of Rehabilitation Medicine Special Interest Group on Mild Traumatic Brain Injury.

The diagnostic criteria for mild TBI, prepared by the American Congress of Rehabilitation Medicine Special Interest Group on Mild Traumatic Brain Injury, are shown in Figure 1 [18].

Medical literature characterizing seizure-related head injuries, especially in comparison to non-seizure-related ones, is scarce. Most authors study injuries after seizures generally, without a specific focus on head injury, although the available sources suggest few differences between TBIs in people with epilepsy compared to the general population without the aforementioned condition. People with epilepsy have an increased risk of severe TBI compared to the healthy population—studies suggest a range of 17% up to 50% increased risk of severe TBI as compared to the general population. The underlying cause remains unclear; proposed explanations include the loss of consciousness during seizures, an atonic state, the frailty of the affected population, and antiepileptic drug side effects. TBIs in people with epilepsy are also more repetitive and more frequently fatal when compared to the population without epilepsy [19,20].

## 4. Consequences

Seizure-related head injuries lead to numerous adverse consequences, both acute and long-term. Acute consequences include skin lacerations, concussions, skull fractures, epi- and subdural hematomas, and intraparenchymal hemorrhages. Head injuries in PWE are generally mild [4,21] and, as such, envelop only skin lacerations, other soft tissue damage, or concussions. SRHIs rarely result in more serious complications requiring neurosurgical intervention. In a study conducted by Russell-Jones et al., after analyzing 12,626 epileptic seizures associated with falls, the researchers found only one confirmed skull fracture, one subdural hematoma, and one epidural hematoma [22]. In a study from North India, only two hematomas requiring surgical evacuation were noted in a population of 171 patients with seizure-related injuries [23]. A 2-year longitudinal study of seizure-related injuries in a population of epilepsy patients at the Baylor Comprehensive Epilepsy Center in Houston, TX, USA showed that severe head injuries are rare, and out of 306 studied patients, only one had an intracranial hematoma requiring neurosurgical intervention, and no skull fracture was reported [24]. A study by Desai et al. included 25 out of 702 studied patients with confirmed seizure-related head trauma resulting in eight skull fractures and eight intracranial hematomas, but only four patients eventually required neurosurgical intervention [25].

On the other hand, in the prospective study by Zwimpfer et al. [26], out of 582 patients with head trauma due to falls, 22 were caused due to seizures and resulted in 20 mass lesions, of which five were epidural hematomas and 12 acute subdural hematomas. Of 22 patients with seizure-related head injuries in the aforementioned study, 18 required surgical evacuation of a hematoma. In the study, both the incidence of hematomas (90.9% vs. 39.8%) and the rate of neurosurgical intervention needed (81.8% vs. 32.3%) suggest that PWE are at increased risk of complicated head injury. The authors argue that the difference between their results and other studies may arise from both group selection—their study had significantly more severe trauma in the whole population than in other comparable studies, as many of their patients were referred for neurosurgical care directly from emergency departments/other hospitals—and also from lower rates of head CT performed in other hospitals as compared to the Desai study.

Post-concussion syndrome (PCS) covers a wide spectrum of symptoms occurring after, mostly, mild traumatic brain injuries, which consist of physical, cognitive, and emotional manifestations. Symptoms of a syndrome are often present after concussion and usually resolve on their own within a few days after the trauma, but in some cases may endure leading to persistent PCS. It is also worth noting that it is still up for debate whether PCS is a condition, per se, or a collection of independent conditions often co-occurring after TBI and, as a result, although present in DSM-IV, post-concussion syndrome is absent in the DSM-V classification [5,27]. The epidemiology of the syndrome differs from study to study, ranging from 30 to 80 percent of patients with mild to moderate head injury. A study by Voormolen et al. assessed PCS incidence in a large cohort of patients divided into two subgroups: complicated and uncomplicated mTBIs. An mTBI was defined as a head trauma with GCS 13–15, and these were divided into complicated and uncomplicated cohorts, whether any abnormality in the acquired head CT was present or not. The presence of PCS at 3 months after injury was 46% in the complicated mTBI group and 35% in the uncomplicated; at the 6-month mark, the prevalence decreased to 43% complicated vs. 34% uncomplicated, respectively [28]. The differences between studies might be partially explained by the diagnostic criteria used by the authors; in a study by Karaliute et al., 15 different methodologies for assessing PCS after a mild TBI were compared by applying them to a population of 221 patients with mild TBIs, eventually resulting in the prevalence of PCS ranging from 10% up to 47%, depending on the used method [29]. The prevalence of symptoms for over 1 year is estimated to be up to 15% of patients affected. PCS may clinically present as headaches, fatigue, malaise, dizziness, imbalance, vision disturbances, somatosensory dysfunction, vestibular dysfunction, insomnia, anxiety, emotional lability, irritability, attention deficits, memory impairment, or alcohol intolerance [30]. Being a frequent, chronic sequela of a TBI, with many potential neurological deficits and manifestations, PCS is an important factor in decreased quality of life in people affected by seizure-related head injuries.

There are not enough studies on the impact of SRHIs on the further course of previously diagnosed epilepsy. Assuming the thesis that head injury may be both a cause and a complication of epilepsy should constitute the closing of a positive feedback loop leading to more frequent and more severe epileptic seizures. However, Friedman et al. showed that although recurrent seizure episodes are common in patients with epilepsy, SRHIs do not influence the further course of the disease. SRHIs also do not affect the incidence of seizures [1]. Such findings may seem surprising, as traumatic brain injury is a well-known risk factor for de novo seizures and epilepsy in otherwise healthy patients [7,31].

As many as 60% of epilepsies are caused by structural damage to the brain. One of the causes of structural pathology in the brain may be an mTBI [32] resulting in post-traumatic epilepsy (PTE). PTE is a chronic seizure condition after a brain injury. The risk of developing post-traumatic epilepsy is directly related to the number and severity of the injury or injuries [33]. Post-traumatic epilepsy accounts for approximately 5% of all diagnosed epilepsies and as much as 20% of epilepsies associated with structural brain damage [34]. As such, it would be reasonable to assume that a TBI—by inducing hyperexcitability and epileptogenesis—would eventually aggravate the aforementioned pathological pathways and worsen the course of the disease, yet the statistical data throughout the available studies do not support such a claim. However, as literature on the topic is scarce, further research is needed.

TBIs may lead to a neuroinflammatory reaction that can secondarily damage the blood–brain barrier (BBB), intensifying the inflammatory response, which may be the source of PTE. TBIs can also cause direct damage to the BBB, creating positive feedback that may result in the development of pathological epileptogenic foci.

An increasing amount of evidence suggests that BBB damage is a complex pathophysiologic process in which corrupted angiogenesis, neuroinflammation, altered glial physiology, abnormal leukocyte–endothelial interactions, and hemodynamic changes eventually result in hyperexcitability.

Neuroinflammation plays a crucial role in BBB permeability. Through elevating levels of interleukins-1β, -6, and TNF-α, it may lead to increased permeability of the BBB and facilitate the movement of peripheral cytokines into the CNS. These cytokines stimulate receptors in the cerebrovascular compartment, which in turn increases the production of cytotoxic substances compromising BBB integrity even further at the cellular level. Direct damage to brain tissue disrupts the BBB, allowing for an influx of immune cells such as leukocytes, macrophages, and neutrophils. NF-κB translocates to microglial nuclei, transforming them into an activated microglia phenotype. This stimulates cellular proliferation and the release of inflammatory mediators such as chemokines, cytokines, reactive oxygen species (ROS), and nitric oxide synthase (NOS). Macrophages, primarily through phagocytosis, take part in the active removal of defective cells and debris in affected areas. However, in some cases instead of initiating repair mechanisms, they may further damage brain tissue. The course of events depends primarily on their level of activity and the expression of signaling molecules. The release of lactate from astrocytes contributes to water retention and osmotic influx of water into cells, resulting eventually in edema. Leaky BBB connections can lead to the migration of iron ions, which can contribute to neuronal hyperactivity. Then, an excessive accumulation of glutamate and aspartate neurotransmitters occurs as a result of leakage from previously damaged neurons or insufficient reuptake by astrocytes. At a later stage, these molecules lead to the activation of NMDA and AMPA receptors located on postsynaptic membranes, which consequently lead to an influx of calcium ions. The increase in Ca^2+^ leads to the production of ROS and the activation of calpains. Adenosine triphosphate (ATP) underproduction by damaged or defective mitochondria eventually leads to an ATP deficit, which results in significant malfunction of Na^+^/K^+^ pumps, activation of Ca^2+^ channels, and further ROS/NOS production. Cytochrome C released from cells into the cytosol activates cell death pathways through caspase pathway proteins [35].

Another long-term consequence of SRHIs worth analyzing may be the impact of repeated head injuries on cognitive abilities. As shown above, the vast majority of SRHIs can be classified as mTBIs. In a 2023 publication, Woods et al. [36] showed in a study of 15,764 patients that mTBIs have a long-term negative impact, primarily on attention and executive functions and, to a lesser extent, on data processing speed and working memory. This study also showed that the severity of brain damage during trauma is directly proportional to the level of deterioration of basic cognitive processes. At the same time, this study showed a steady deterioration in cognitive functions over 4 years of observation. After the initial decline in cognitive function as a result of an mTBI, the cognitive deficit did not worsen over time.

## 5. SRHI Emergency Department Management

Management of a seizure-related head injury, aside from the thorough physical examination and medical history that every TBI patient requires, depends substantially on the severity of the trauma. Severe traumatic brain injuries require a systemic approach to avoid any secondary injury that can be caused by hypoxia, hypoglycemia, or hypotension, and immediate neuroimaging followed by a neurosurgical consult [8]. The most common imaging modality used to assess brain injury is non-contrast head CT, as it is readily available in hospitals and can identify most of the TBI acute complications such as intracranial hematomas, brain edema, or skull fractures that may require neurosurgical intervention. Although non-contrast head CT has good sensitivity for acute bleeds and fractures, its usefulness in detecting diffuse axonal injury (DAI), parenchymal contusion, or secondary ischemic changes due to cerebral edema and/or intracranial hypertension is limited [37]. In some cases, MRI might be more useful, e.g., in cases of persistent neurological symptoms without any clear findings on the head CT. When assessing whether a patient with TBI has ongoing seizure activity, two useful modalities arise: functional magnetic resonance imaging (fMRI) and optical modalities such as near-infrared spectroscopy (NIRS) [38]. Functional MRI can detect local blood-oxygen-level-dependent (BOLD) signals correlating to brain activity in the area. This allows for the detection of areas of increased, rapidly changing activity, which may indicate epileptic activity. Optical modalities like NIRS can measure local oxy- and deoxyhemoglobin concentrations from changes in infrared wavelength absorption. Although limited only to the surface area, NIRS is a cheaper, more mobile, and easier-to-obtain modality than fMRI. These functional neuroimaging modalities may be found useful in differentiating types of epilepsy, e.g., temporal lobe epilepsy or absence epilepsy. Although fMRI and NIRS are currently not present in routine clinical workups, especially in ED settings, research results seem promising.

As the protocol seems unambiguous when severe injury is considered, with less severe injuries, the optimal clinical management is often unclear. In cases of mild SRHIs, the most important question is whether to perform head CT. The Eastern Association for the Surgery of Trauma recommends that every patient presenting with suspected traumatic brain injury should have a brain CT scan performed if possible [39]. In cases of limited CT resources, the authors suggest using standardized criteria (such as the Canadian CT head rule or the New Orleans Criteria) to determine patients needing a scan. The most popular tool used to determine clinical indication to perform head CT is the Canadian CT head rule (CCTHR). The CCTHR can be used in patients who experience a traumatic brain injury, have a Glasgow Coma Scale score of at least 13, are above the age of 16, have no coagulopathy nor are using anticoagulants, and have no visible open skull fracture [40].

Under the CCTHR, patients with mild TBIs should be given a head CT if at least one of the following is met:

- GCS score less than 15 after 2 h,
- Suspected open or depressed skull fracture,
- Suspected basal skull fracture,
- At least two episodes of vomiting,
- Age 65 or older,
- Amnesia before impact lasting for at least 30 min,
- Dangerous mechanism of injury, e.g., motor vehicle accident.

The first five criteria are considered high risk, as they correspond with potential neurosurgical intervention, and the last two are considered medium risk, corresponding to the potential finding of brain injury on CT. Patients with a mild TBI and a negative brain CT scan can be discharged from ED if they have no other injuries or medical issues requiring hospitalization, without a neurosurgical consult. Patients using anticoagulants such as warfarin need prolonged clinical observation, even with normal head CT results, because of the increased risk of interval intracranial hemorrhage.

As seizure-related brain injuries come with many potential short- and long-term consequences, which may potentially lead to disabilities or cognitive decline, and the existence of troublesome areas such as the lack of concise and standardized indications for head CT in mTBIs, there is a dire necessity for a distinct protocol for the management of seizure-related brain injuries in emergency department settings, especially considering the resource limitations of public healthcare systems.

Analyzing a number of publications, it seems that from the perspective of an emergency department physician, the treatment of a patient with TBI is the same, regardless of the etiology of the injury. This should first of all be based on the symptoms presented by the patient and his clinical parameters. At later stages of hospitalization, during further evaluation, neuroimaging should be planned accurately according to both the background of injury and possible etiology to select the imaging modality that would be the most accurate to assess suspected complications that did not require medical attention immediately in the emergency department, and comorbidities related to the injury.

## 6. Conclusions

Seizure-related head injuries are an important clinical problem frequently encountered in emergency departments. SRHIs are one of the most frequent types of injury reported after seizures. SRHIs can lead to various acute complications that in some cases may require hospitalization and neurosurgical intervention. Long-term complications and cognitive decline after injury might be present, eventually implying a negative impact on a patient’s quality of life. Despite being frequent and clinically important, there are still no widely accepted, uniform recommendations for the management of patients with SRHI. There are still troublesome areas such as a lack of standardized indications for head CT in the case of mild TBIs, which make up the majority of SRHIs, with a few different sets of criteria aimed at helping clinicians with decisions. As such, a concise and standardized protocol for the management of seizure-related head injuries in emergency departments is worthy of consideration.
